# Computational Design of Peptide Ligands for Ochratoxin A

**DOI:** 10.3390/toxins5061202

**Published:** 2013-06-21

**Authors:** Meike Heurich, Zeynep Altintas, Ibtisam E. Tothill

**Affiliations:** Cranfield Health, Cranfield University, Cranfield, Bedfordshire MK43 0AL, England, UK; E-Mail: zeynep.altintas@cranfield.ac.uk (Z.A.)

**Keywords:** ochratoxin A, mycotoxins, peptide, computational modelling, surface plasmon resonance, biosensor

## Abstract

In this paper, we describe a peptide library designed by computational modelling and the selection of two peptide sequences showing affinity towards the mycotoxin, ochratoxin A (OTA). A virtual library of 20 natural amino acids was used as building blocks to design a short peptide library against ochratoxin A template using the *de novo* design program, LeapFrog, and the dynamic modelling software, FlexiDock. Peptide sequences were ranked according to calculated binding scores in their capacity to bind to ochratoxin A. Two high scoring peptides with the sequences N'-Cys-Ser-Ile-Val-Glu-Asp-Gly-Lys-C' (octapeptide) and N'-Gly-Pro-Ala-Gly-Ile-Asp-Gly-Pro-Ala-Gly-Ile-Arg-Cys-C' (13-mer) were selected for synthesis from the resulting database. These synthesized peptides were characterized using a microtitre plate-based binding assay and a surface plasmon resonance biosensor (Biacore 3000). The binding assay confirmed that both *de novo* designed peptides did bind to ochratoxin A *in vitro*. SPR analysis confirmed that the peptides bind to ochratoxin A, with calculated *K*_D_ values of ~15.7 μM (13-mer) and ~11.8 μM (octamer). The affinity of the peptides corresponds well with the molecular modelling results, as the 13-mer peptide affinity is about 1.3-times weaker than the octapeptide; this is in accordance with the binding energy values modelled by FlexiDock. This work illustrates the potential of using computational modelling to design a peptide sequence that exhibits *in vitro* binding affinity for a small molecular weight toxin.

## 1. Introduction

The control of common food contaminants, like mycotoxins, is creating a strong demand for analytical methods that permit their rapid and sensitive detection at established regulatory limits. Mycotoxins are toxic secondary metabolites produced by various fungi (*Aspergillus*, *Penicillium* and *Fusarium*) in a wide variety of foods, such as maize, coffee beans, cocoa or soy beans, as well as meat, milk and grapes, but also beverages, like coffee, beer, grape juice or wine [1,2,3,4,5]. Occurrence and growth of mycotoxins depends on both environmental and food manufacturing conditions [6,7]. The diverse chemical structures of mycotoxins and their differing physical properties can exhibit a wide array of biological effects on mammalian systems, e.g., genotoxic, teratogenic, mutagenic, embryogenic or estrogenic [8], and some show immunosuppressive activity [9,10]. The mycotoxin, ochratoxin A, has been considered by the International Agency for Research on Cancer to be possibly carcinogenic (group 2B) for humans [11,12,13,14,15,16]. There is a growing need to monitor ochratoxin A in food and beverages according to the EU maximum permitted level of 2.0 μg L^−1^ of ochratoxin A [17]. Analytical methods for the determination of ochratoxin A in wine are generally based on thin-layer chromatography (TLC) or high-performance liquid chromatography (HPLC) [18,19,20]. However, these techniques either suffer from inadequate sensitivity, due to the lack of a sensitive universal detector for mycotoxins [21], or are expensive and time-consuming [5,22]. Although chromatography-based methods are sufficiently sensitive and accurate, immunoassay-based test kits are a good alternative method for high-throughput analysis, where antibodies are used as the recognition elements, providing the specificity and sensitivity required for low level toxin detection. Antibody against ochratoxin A has been successfully developed and employed in immunoassays [23,24,25] and an immunoaffinity-based electrochemical sensor [26,27]. While these methods are attractive, immunoassays are not re-usable, have a limited storage time and, in some cases, antibodies may show cross-reactivity with other compound in the food matrix. Whereas the affinity sensor development in our previous work focused on optimization of detection sensitivity with regards to the sensor platform using a disposable CM-modified gold electrode and antibodies as the sensing molecule [26], there is still a need for a sensitive, specific and bio-stable sensing receptor for ochratoxin A, which can be easily and cost-effectively produced as a viable alternative for antibodies. 

Replacing natural biomolecules with artificial receptors or biomimics has become an attractive area of research in recent years [28,29]. The advantages of using these molecules are that they are robust, more bio-stable and cost-effective to produce and can be modified easily to aid immobilization and to add labels for detection. Molecular recognition by peptides is known for a number of biochemical processes, such as signal transduction, metabolism, cell growth and immune defence, and is based on several non-covalent interactions (e.g., H-bridge-bonding, salt-bridges, hydrophobic and van der Waals interactions) [30]. Peptide receptors have many advantages over antibodies in that specific peptides can be obtained for virtually any target, even those that are toxic or have low immunogenicity. Peptides can be chemically synthesized, offering a wide variety of targeted modifications, such as fluorescent or affinity tags. Due to their small molecular weight, they are more stable in a wide range of buffer solutions and less prone to activity loss [31,32]. 

Recent literature described a combinatorial approach to select peptides that have been specifically synthesized to target specific molecules, including the mycotoxins, ochratoxin A and aflatoxin B [33,34,35]. Combinatorial peptide libraries, which consist of up to a million synthetic peptides, are complex enough to offer unique binding sites that can be screened for peptide receptors with improved selectivity for a specific target molecule. In contrast to combinatorial chemistry, which is based on the screening of a large amount of compounds that were randomly synthesized, the structure-based receptor design is built on the known target structure. Computational modelling is a technique of representing molecular structures numerically and simulating their behaviour with quantum equations and classical physics. Computational modelling programs, such as SYBYL [36], allow researchers to generate and present molecular data, including geometries (bond lengths, bond angles, torsion angles), energies (e.g., heat of formation, activation energy), electronic properties (moments, charges, ionization potential, electron affinity), spectroscopic properties (vibrational modes, chemical shifts) and bulk properties (volumes, surface areas, diffusion, viscosity) [37]. Molecular modelling software and searching algorithms are traditionally applied in drug design [38]. This technique has been successfully employed in our lab to gain a selection of synthetic receptors based on molecular imprinting technology (MIP) that were able to interact specifically with the ochratoxin A template for use in solid-phase extraction chromatography [39,40]. 

Here, we present the application of computational modelling for a peptide ligand designed for ochratoxin A binding. Using molecular modelling software for the development and screening of peptide sequence libraries around a known small molecular weight target structure is a novel approach. Computationally-derived peptides were synthesized, and the *in vitro* binding interaction with ochratoxin A was investigated using solid-phase binding assays and binding affinity determined by a surface plasmon resonance biosensor (SPR, Biacore 3000). 

## 2. Results and Discussion

### 2.1. Computational Modelling

**Figure 1:**
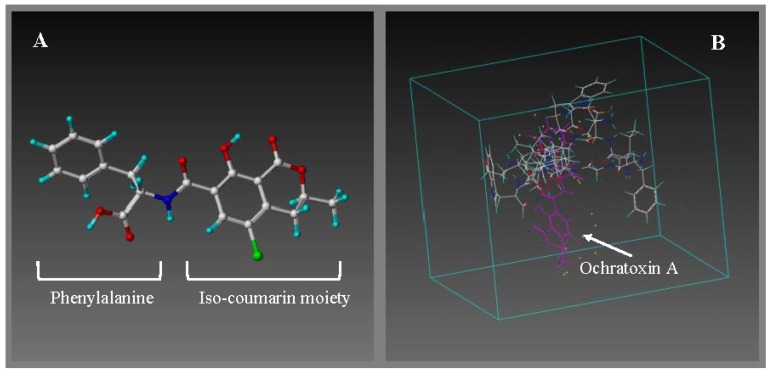
(**A**) Minimized and annealed structure of the ochratoxin A template (shown as stick and ball); (**B**) The generation of new peptide compounds begins with a pool of potential monomers (amino acids) and a virtual cavity (shown as a box) in which to place them with the target molecule, *i.e*., ochratoxin A. This virtual screening process is called “electrostatic screening”. The ochratoxin A template is shown in purple interacting with random amino acid monomers (stick and ball) was screened using the LeapFrog design tool.

Modelling the binding of ochratoxin A interaction with individual amino acid monomers was performed to determine the binding score of each interaction (Figure 1). The monomers included in the virtual library screening for ochratoxin A were all natural amino acids. The results for each amino acid are compared on the basis of their binding scores, resulting in a table ranking them according to their binding scores, expressed as a binding energy value in kcal/mol. Descending binding energy values of the amino acids are proportional to the ascending binding score, *i.e*., the most likely binding interaction with ochratoxin A. 

Table 1 depicts the binding energies for amino acid monomers interacting with the ochratoxin A template. The top five scoring amino acids interacting with ochratoxin A are phenylalanine (F) > proline (P) > valine (V) > isoleucine (I) > leucine (L), all containing apolar side chains and exhibiting hydrophobic characteristics. The highest binding score was seen with phenylalanine, which contains a water-insoluble aromatic ring, which is interesting, since the ochratoxin A structure contains a l-β-phenylalanine moiety also (shown in Figure 1A). 

**Table 1:** Binding energies for amino acid monomers modelled with ochratoxin A (shown in descending order from the top down).

Amino acid	Polarity	Binding energy (kcal mol^−1^)
Phenylalanine **Phe**)	Apolar	−33.52
Proline (**Pro**)	Apolar	−32.10
Valine (**Val**)	Apolar	−30.93
Isoleucine (**Ile**)	Apolar	−30.37
Leucine (**Leu**)	Apolar	−28.94
Cysteine (**Cys**)	Polar (uncharged)	−28.67
Tyrosine (**Tyr**)	Polar (uncharged)	−27.29
Methionine (**Met**)	Apolar	−26.33
Threonine (**Thr**)	Polar (uncharged)	−25.55
Tryptophan (**Trp**)	Apolar	−22.71
Alanine (**Ala**)	Apolar	−21.87
Glutamate (**Glu**)	Polar (negatively charged)	−20.63
Aspartate (**Asp**)	Polar (negatively charged)	−19.86
Asparagine (**Asn**)	Polar (uncharged)	−13.27
Lysine (**Lys**)	Polar (positively charged)	−11.72
Histidine (**His**)	Polar (positively charged)	−10.06
Glutamine (**Gln**)	Polar (uncharged)	−6.43
Arginine (**Arg**)	Polar (positively charged)	−5.65
Serine (**Ser**)	Polar (uncharged)	−5.23
Glycine (**Gly**)	Polar (uncharged)	−1.89

The *de novo* design of peptide receptors was carried out using the molecular design software, LeapFrog, in DREAM-mode (default “Tailor” option), proposing new molecules (short peptide sequences) from the 20 amino acid library shown in Table 1. The highest scoring peptide sequences interacting with ochratoxin A are shown in Table 2.

**Table 2:** Binding energy of top five highest scoring peptide sequences (shown in descending order from the top down) interacting with ochratoxin A generated from LeapFrog in DREAM mode.

Sequence	Binding energy (kcal mol^−1^)
Ile-Gly-Ala	−44.55
Ile-Gly-Ala-Pro	−44.54
Ile-Gly-Ala-Gly	−40.05
Ile-Gly-Ala-Cys	−38.56
Ile-Gly-Ala-Pro-Ala	−37.47

Short peptide sequences were generally between three and five amino acid residues in length. The top five highest scoring peptide sequences repetitively contained the amino acids isoleucine (I), glycine (G), alanine (A) and proline (P). The inclusion of proline and isoleucine in the peptide sequence was expected, since these amino acid monomers resulted in high binding scores when modelling the monomer interactions, as seen in Table 1. Alanine scored average; however, glycine showed the lowest binding score. Glycine has no ionizable side groups; thus, it cannot be involved in any binding event when it is located within a peptide, and it is mainly involved as a structural spacer.

The library of 20 natural amino acid monomers was screened modifying the LeapFrog “Tailor” option in “relative move frequencies” in their “join” (default 2, modified 6) and “bridge” (default 2, modified 0) parameters. The “Tailor” option allows LeapFrog to combine amino acids into short peptide chains through the “Join” parameter. LeapFrog produced a database of peptide sequences of 3–6 amino acids in length. The highest scoring peptide sequences in descending order interacting with ochratoxin A are shown in Table 3. 

**Table 3:** Binding energies for the highest scoring peptide sequence (shown in descending order from the top down) interacting with ochratoxin A generated from LeapFrog with modified “Join” and “Bridge” parameters.

Amino acid sequence	Binding energy (kcal mol−1)
Pro-Ser-Ile-Val-Glu	−46.45
Ile-Gly-Ala	−44.55
Ile-Gly-Ala-Pro	−44.54
Cys-Ser-Ile-Val-Glu	−42.13
Ile-Gly-Ala-Pro-Ala	−37.47
Cys-Gly-Pro-Ala-Gly-Ile	−31.85
Ser-Pro-Ala-Gly-Ile	−31.56

The highest scoring peptide sequences repetitively contained the amino acids isoleucine, glycine, alanine and proline, as well as valine, glutamate and serine. The introduction of proline and isoleucine in the peptide sequences was expected, since these amino acid monomers resulted in high binding scores, previously as monomers and in short sequences (Table 1, Table 2). Valine is another high scoring amino acid when modelling the monomer interaction, whereas glutamate scored as average as alanine and serine as low as glycine. Interestingly, the majority of these amino acids are apolar, which indicates the involvement of hydrophobic interactions; however, this also means that these peptides are difficult to dissolve in aqueous solution. 

To enhance the affinity of the peptide interaction with ochratoxin A, as well as improve the solubility of the final peptide to be synthesized, the peptide sequences obtained from the LeapFrog database (Table 3) were manually modified. Short peptide sequences were first dimerized to enhance affinity and, due to the nonpolar nature of the ochratoxin A ligand, charged or polar amino acids were carefully chosen for incorporation into the receptor to provide a second representative range of polar, charged and hydrophobic monomers. The molecular dynamics of these peptide receptors with ochratoxin A was modelled using FlexiDock, which calculates the binding interaction assuming the high flexibility of the peptide around its template ochratoxin A. The resulting database of high scoring peptide sequences contained a mixture of unmodified and modified (dimerized, charged) peptide sequences, as shown in Table 4. 

**Table 4:** List of 11 high scoring peptides obtained with FlexiDock shown in descending order from the top down.

Peptide sequence	Binding energy (kcal mol^−1^)
Gly-Pro-Ser-Ile-Val-Glu-Cys	−17.24
Pro-Ser-Ile-Val-Glu-Pro-Ser-Ile-Val-Glu-Cys	−16.72
Ser-Pro-Ala-Gly-Ile	−16.07
**Cys-Ser-Ile-Val-Glu-Asp-Gly-Lys**	**−14.90**
Cys-Gln-Ile-Val-Glu-Pro-Gln-Ile-Val-Glu	−14.63
Cys-Phe-Asp-Pro-Ala-Gly-Ile-Lys	−14.25
Cys-Phe-Asp-Ala-Pro-Ala-Gly-Ile-Lys	−13.08
Pro-Ser-Ile-Val-Glu	−12.48
**Gly-Pro-Ala-Gly-Ile-Asp-Gly-Pro-Ala-Gly-Ile-Arg-Cys**	**−11.81**
Gly-Ser-Pro-Ala-Gly-Ile-Gly	−11.78
Cys-Gly-Pro-Ala-Gly-Ile	−8.72

The resulting binding energy values obtained by FlexiDock are not identical to the Tripos force field and the LeapFrog application and need to be seen independently of earlier simulations, because different force field terms were used and a site-point matching score was included in the FlexiDock calculation. The FlexiDock database was used to compare the binding energies for modified and unmodified sequences. Notably, the binding energy values for both modified and unmodified peptides were similar, implying that the modifications with charged amino acids did not decrease the binding affinity of the peptide to ochratoxin A; however, dimerization did not seem to have a significant effect on binding energy values either.

**Figure 2:**
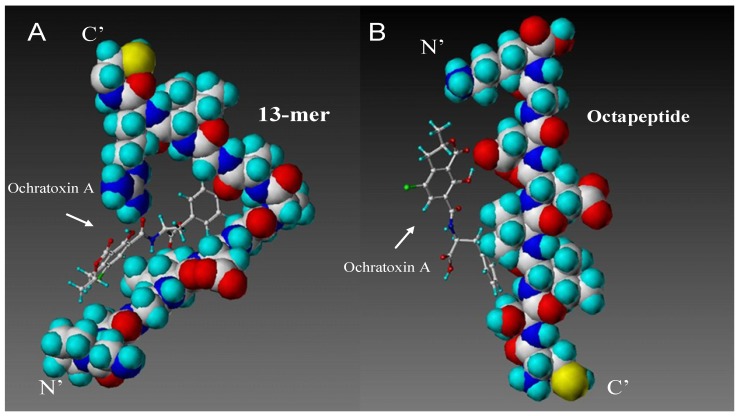
Final result of *de novo* designed peptide sequences shown interacting with ochratoxin A. The [*N*'-Gly-Pro-Ala-Gly-Ile-Asp-Gly-Pro-Ala-Gly-Ile-Arg-Cys-*C*'] peptide (**A**, left) and the [*N*'-Cys-Ser-Ile-Val-Glu-Asp-Gly-Lys**-***C*'] peptide (**B**, right) sequence are seen as space-filled, ochratoxin A as stick and ball structures. Ochratoxin A mainly interacts with the peptide backbone of the *N*-terminal end of the 13-peptide (B) and the central region of the Octamer peptide backbone.

From this database, two high scoring sequences were chosen for further analysis, a 13-peptide with the sequence (*N*'-Gly-Pro-Ala-Gly-Ile-Asp-Gly-Pro-Ala-Gly-Ile-Arg-Cys-*C*') (Figure 2A), which is a dimer derived from the basic sequence (Pro-Ala-Gly-Ile) listed in Table 2 as the second best score. This dimer is separated by negatively charged aspartate and glycine to allow for flexibility, as well as *C*'-terminal charged arginine for solubility and the *N*'-terminal cysteine tag for immobilization purposes. The second sequence is an octapeptide (*N*'-Cys-Ser-Ile-Val-Glu-Asp-Gly-Lys**-***C*'), as seen in Figure 2B, which has been derived from the top score sequence, Ser-Ile-Val-Glu (Table 3). This sequence was modified by introducing an *N*'-terminal cysteine and a *C*'-terminal negatively charged aspartate, as well as glycine for flexibility. 

Both selected peptide sequences have the amino acid isoleucine in common, which showed a very high binding score when interacting with ochratoxin A (Table 1). Isoleucine has no ionizable groups and, therefore, cannot be taking part in hydrogen bonding. Since isoleucine is very hydrophobic, it is likely that it attracts molecules, such as ochratoxin A, through hydrophobic interactions. Hydrophobicity is one of the major forces in ligand recognition. As the hydrophobic side-chains of an amino acid sequence and a hydrophobic ligand come together, there is a favourable increase in the entropy of the system as the solvent (water) molecules, which were previously in an ordered shell around the exposed hydrophobic surface, become disordered, as well as an energetic contribution as unfavourable apolar-polar interactions are replaced with more favourable homotypic interactions [41,42,43]. It seems the binding interaction of ochratoxin A with the designed peptides is established by electrostatic/hydrophobic interactions rather than hydrogen bonding. 

The two peptide sequences were then synthesized and mass spectroscopy was applied to obtain the molecular weights of the peptides. The Medical Research Council (MRC, UK) submitted molecular weights for the octapeptide at 834 g mol^−1^ and for the 13-mer peptide, 1183 g mol^−1^. Both peptides were water soluble.

### 2.2. Binding Assay of Ochratoxin A-HRP to the 13-mer and Octapeptide

**Figure 3:**
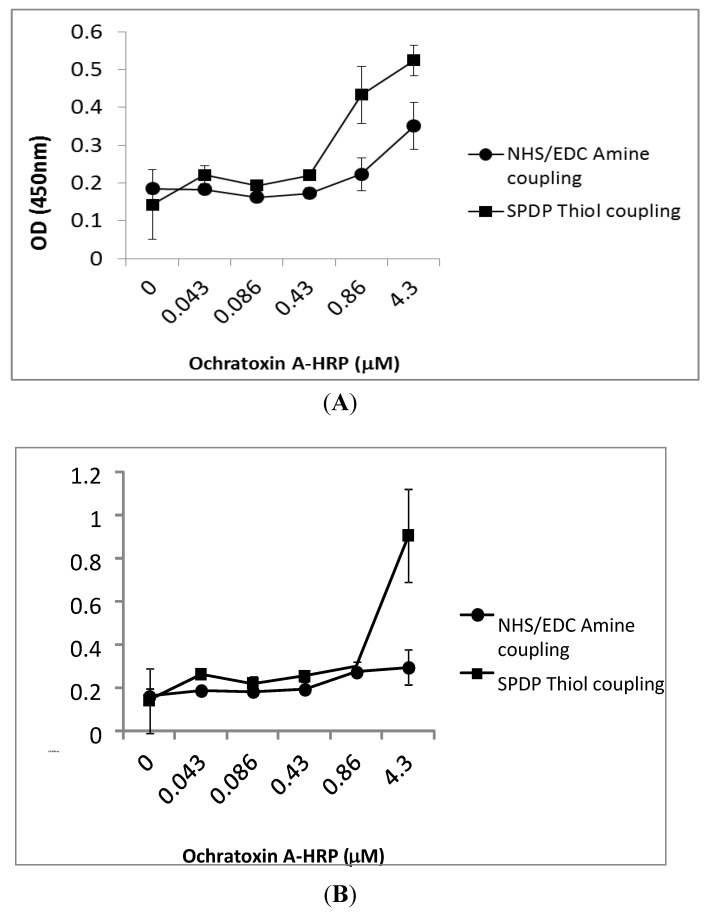
Capacity of the ochratoxin A-HRP to bind to the octapeptide and 13-mer peptide. The interaction between serial dilutions of HRP-conjugated ochratoxin A and octapeptide (**A**) and 13-mer peptide (**B**) immobilized to functionalized microtitre plates via amine coupling (●) and thiol coupling (■) is expressed as absorbance at A 450 nm *versus* ochratoxin A-HRP concentration (µM). Error bars illustrate the mean and standard deviation of multiple experiments.

Solid phase binding assay provides a platform to test the binding capacity of the designed peptides to ochratoxin A *in vitro*. Each peptide sequence was immobilized to the surface of a functionally modified (R2-NH) microtitre plate via either amine coupling using *N*-hydroxysuccinimide (NHS)/*N*-(3-dimethylaminopropyl)-*N*'-ethylcarbodiimide (EDC) coupling chemistry or thiol coupling using a heterobifunctional crosslinker (*N*-Succinimidyl 3-(2-pyridyldithio) propionate (SPDP)) employing the terminal cysteine residues of the peptide sequences to allow for site-directed surface attachment. Enzyme-labelled ochratoxin A-HRP was added in a concentration-dependent manner to the immobilized peptides, and the absorbance signal (blank subtracted) was compared for both peptides and immobilization techniques, as shown in Figure 3. 

The absorbance signal confirmed that ochratoxin A-HRP binds to both amine- and thiol-coupled octapeptide (Cys-Ser-Ile-Val-Glu-Asp-Gly-Leu) (Figure 3A). However, much higher binding signals were obtained with the peptide immobilized using thiol chemistry. The 13-mer peptide **(**Gly-Pro-Ala-Gly-Ile-Asp-Gly-Pro-Ala-Gly-Ile-Arg-Cys**)** showed good binding capacity to ochratoxin A-HRP when thiol coupled, which was decreased when immobilized using amine coupling chemistry. The results suggest that both peptides bind ochratoxin A (Figure 3B).

### 2.3. SPR Analysis to Determine Binding Affinity

**Figure 4:**
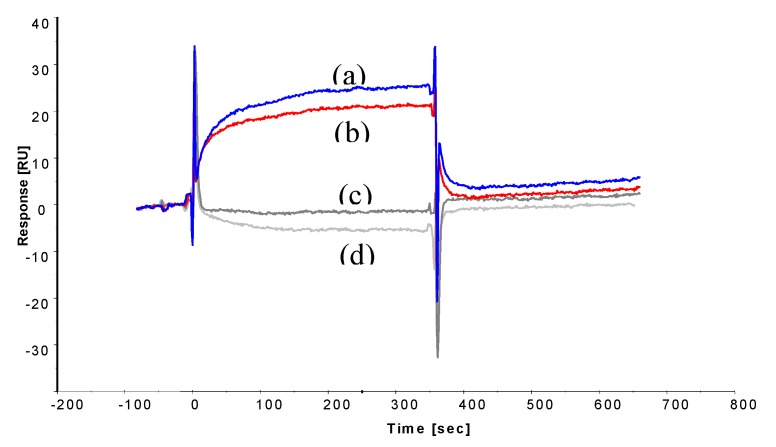
Sensorgrams displaying binding curves of 100 mg L^−1^ (0.15 μM) ochratoxin A-BSA (reference subtracted) to immobilised peptides, (**a**) 13-mer peptide GPAGIDGPAGIRC (blue) and (**b**) octapeptide CSIVEDGL (red); and 100 mg L^−1^ BSA alone (negative control) binding to (**c**) 13-mer peptide (dark grey) and (**d**) octapeptide (light grey).

To determine affinities for the peptide binding interaction, the peptides were immobilized on two adjacent flow cells onto a CM5 Biacore chip to a level of 251 RU (octapeptide) and 227 RU (13-mer peptide). This was in order to study the binding interaction between the peptides and ochratoxin A using a Biacore 3000. A commercial ochratoxin A-BSA conjugate was used as the analyte to obtain a significant signal response for a binding event. Initially, one saturating concentration of ochratoxin A-BSA conjugate (100 mg L^−1^) was injected over each immobilized peptide ligand to confirm binding (Figure 4). 

**Figure 5:**
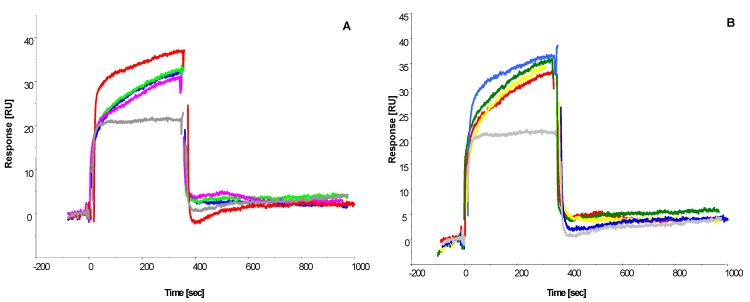
Sensorgrams showing the binding interaction of immobilized (**A**) octamer peptide CSIVEDGL and (**B**) 13-mer peptide GPAGIDGPAGIRC with decreasing ochratoxin A-BSA analyte concentration (from top to bottom: 100, 1, 0.1, 0.01 mg L^−1^; equivalent to 15pM–0.15 μM. BSA reference binding is shown in grey.

Injection of BSA alone showed no binding, confirming the binding interaction to be ochratoxin A-dependent. To establish the affinity of the interaction, decreasing ochratoxin A-BSA concentrations were injected onto the immobilized peptides surfaces (Figure 5). 

Figure 4 shows that both the 13-mer and octapeptide bind the ochratoxin A-BSA conjugate and show no non-specific binding to the unconjugated BSA used as the reference, which was subtracted from the signal curves. Immobilization methods, especially via amine coupling, can reduce the activity of the immobilized molecule and, thus, have reduced binding capacity, which can be substantially different from the theoretical binding capacity. This is a common and known problem in binding interaction analysis. However, binding curves on the remaining active peptide molecules are still valid and can be used to determine whether a binding interaction is taking place, especially when compared to a reference surface (which is the case here) and also determine the binding affinity of an interaction. The sensorgrams show fast on and off rates for both peptides with ochratoxin A interaction, as the baseline was reached almost immediately after the end of ochratoxin A-BSA injection, indicating weak affinity interactions. Those can be advantageous, as there is no need for a regeneration step, and hence, the analysis can be performed in an isocratic buffer environment, which enhances the stability of the peptides and improves the life-time of the receptor surface. 

Figure 5 illustrates the concentration-dependent binding interaction of ochratoxin A-BSA to the octamer peptide (Figure 5A) and the 13-mer peptide (Figure 5B). Both peptides show similar binding curves and response units relative to ochratoxin-BSA concentration. 

Due to the 1:3 to 1:6 molecular distribution of 3–6 mol ochratoxin A bound per mol BSA (manufacturer statement), the binding interaction does not necessarily follow 1:1 binding interaction stoichiometry, as all 3–6 ochratoxin A molecules can theoretically partake in a binding event. The Biacore evaluation software does not allow affinity calculations for multiple analytes; thus, we chose the standard 1:1 Langmuir binding model to calculate an estimated affinity for the interaction. 

The 13-mer peptide exhibited an estimated binding strength to ochratoxin A with a *K*_D_ of 15.7 μM, and the octapeptide peptide resulted in a similar *K*_D_ of 11.8 μM, which is categorized as weak binding affinity. In contrast, a commercial ochratoxin A-specific antibody was tested and showed relatively high affinity and slow off rates, indicated by a *K*_D_ of 3 nM (data not shown) when immobilized and subjected to the same ochratoxin A-BSA concentrations and conditions. The affinity of the artificial peptide receptors corresponds well with the binding energy values derived from modelling the interaction using FlexiDock (Table 4). The 13-mer peptide has about 1.3-times weaker binding affinity to ochratoxin A than the octapeptide, which is mimicked in the 1.3-times higher binding energy value calculated by FlexiDock (13-mer: −11.81 kcal/mol; Octamer: −14.9 kcal/mol). In conclusion, the results were similar to those achieved in the previous assay format. A competitive assay with free ochratoxin A and ochratoxin A-BSA on the SPR sensor immobilized peptides will need to be conducted to further study the binding interaction of the peptides to ochratoxin A. 

In comparison, the affinity of ochratoxin A binding non-covalently to its natural ligand (human) albumin shows both ionic and hydrophobic forces, [44] and it has been shown that high affinity binding sites can give *K*_D_ ≈ 19 nM, while weak affinity sites show *K*_D_ ≈ 1 µM [45]. The high affinity binding site matches the same affinity range (nM) of a commercial ochratoxin A antibody for ochratoxin-BSA, whereas the weak affinity binding site falls within the same affinity (μM) range as the computationally designed peptides reported in this work. Other peptide receptors for mycotoxins selected from combinatorial peptide libraries also showed μM affinities within the same range, such as an ochratoxin A-specific peptide selected from a combinatorial peptide-phage display library with the sequence *N*'-Ser-Asn-Leu-His-Pro-Lys-*C*', resulting in *K*_D_ ≈ 2.9 μM. Furthermore, Tozzi *et al*. created a combinatorial library with the lead tetrapeptide, Leu-Leu-Ala-Arg-NH_2_, with binding constants *K*_D_ ≈ 8.3 μM and 3.4 μM for aflatoxins B1 and B2, respectively [35]. This further shows that the affinity of our computationally designed peptides matches the same range of those selected from combinatorial libraries, validating computational library design and screening as a good alternative to *in vitro* techniques. We further believe that these peptides, after careful cross-interaction studies and further optimization and characterization, could be employed as a diagnostic tool for ochratoxin A.

## 3. Experimental Section

### 3.1. Materials and Reagents

The ochratoxin A-BSA conjugate, *N*-Succinimidyl 3-(2-pyridyldithio) propionate (SPDP) and cysteine were purchased from Sigma Aldrich Ltd., UK. Acetate buffer and glycine were from Fluka (Sigma-Aldrich Ltd., Dorset, UK). The CM5 sensor chips, HEPES buffered saline (HBS-EP), 1 M ethanolamine-HCl, EDC (*N*-(3-dimethylaminopropyl)-*N*'-ethylcarbodiimide) and NHS (*N*-hydroxysuccinimide), as well as the BIAcore 3000™ used for the analysis were from BIAcore (Uppsala, Sweden). The ready-made 1-Step™ Ultra TMB (3,3',5,5'-tetramentylbenzidine) solution was from Pierce (Pierce Inc., 3747 N. Meridian Road, P.O. Box 117, Rockford, IL 61105, USA). Nunc™CovaLink™ NH-microtiter plates were from NunBrand, Denmark. Peptide sequences have been synthesized and verified by mass spectroscopy by the Medical Research Council (MRC) at Imperial College, London. 

### 3.2. Computational Modelling

The workstation employed to simulate receptor-ligand interactions was a Silicon Graphics Octane running the IRIX 6.6 operating system. It was configured with two 195 MHz reduced instruction set processors, 712 MB memory and a 12 GB fixed drive. This system was used to execute the software package, SYBYL 6.9/7.0 (Tripos Inc., St. Louis, MO, USA). Computational design was performed as follows. A structural model of the ochratoxin A template was drawn according to SYBYL tutorial “small molecule sketching”. The structure was refined using molecular mechanics by applying energy minimization with the MAXIMIN2 command. A simulated annealing process was then applied to obtain conformational energies lower than the minimum of energy found by energy minimization to ensure a conformation that is as close to nature as possible (Figure 1A). Annealing conditions were fixed as 700–200 K for 1000 fs each and run for 1000 cycles [39]. The TRIPOS force field was applied for energy calculations. The dielectric constant corresponded water conditions, and the termination used was “Gradient” with a cut-off value of 0.001 kcal mol^−1^ at a maximum of 1000 iterations. Structures were charged using the Gasteiger-Hückel charges. For peptide receptor design, a molecular library containing 20 natural amino acids was used as monomers. The LeapFrog genetic algorithm was applied to screen each functional monomer of an amino acid library for its possible interaction with the ochratoxin A template (Figure 1B). LeapFrog is an algorithm that allows for evaluations of new ligand structures on the basis of calculating binding scores expressed as an energy value in kcal/mol. This is calculated using electrostatic screening by trying repeatedly distinct amino acid monomers (one each time) in different positions of the ochratoxin A template and then either keeping or discarding the results depending on the calculated binding score of the total contributions from steric, electrostatic and hydrogen bonding interactions (Tripos Association, St. Louis, MO, USA). LeapFrog was applied in DREAM mode and activated for different length of runs (100,000 to 1,000,000 iterations). Amino acid monomers were modified by adding “active hydrogens” for localizing the site of interaction, which facilitates peptide bond formation. The input data was set on “peptide” mode; this allows the receptor to be built by placing amino acid monomers and linking them together to produce the peptide sequence. The binding scores were saved in a database and evaluated mainly on their empirical binding energy. Binding energy, as calculated in LeapFrog, has three major components: steric and electrostatic enthalpies of binding process calculated using the Tripos force field, cavity desolvation energy and ligand desolvation energy. The peptide sequences giving the highest binding score (lowest binding energy value) were selected as candidates for further screening using the ligand-receptor docking module, FlexiDock (Tripos Association, St. Louis, MO, USA).This is a software tool that calculates the binding interaction assuming the high flexibility of the peptide receptor around its template (*i.e*., ochratoxin A). The docking simulation was performed using high scoring peptide sequences obtained from LeapFrog, as well as manually modified sequences. The latter was done under the consideration that some structures selected as high scoring by LeapFrog are synthetically difficult to produce. Thus, charged amino acids were added to the structure to influence overall molecular hydrophilicity with the prospect of producing a water soluble peptide. The final peptide design from FlexiDock contained a LeapFrog-derived template sequence, which was modified in terms of solubility and ease of immobilization in future binding assays (by addition of a cysteine residue).

### 3.3. Solid Phase Binding Assay

To test the initial binding of the peptides with ochratoxin A, the peptides were immobilized to a (NH)-functionally modified polystyrene microtitre plate via: (1) amine-coupling using a NHS/EDC linker and (2) thiol-coupling using SPDP, a hetero-bifunctional cross-linker reagent with amine and sulfhydryl groups. 

Amine coupling was performed as follows: 100 mg L^−1^ of each peptide solution in 10 mM carbonate buffer pH 9.6 was incubated on the NH-wells for 30 min, then 200 mM EDC and 50 mM NHS (1:1 *v*/*v*) were added and incubated for one hour. The plates were washed using PBST and blocked using 0.1 M ethanolamine for 30 min. Thiol coupling was performed via the immobilization of 1.5 mg mL^−1^ SPDP in phosphate buffer (PBS) pH 7.4 incubated for 30 min at room temperature. The peptides were added at 100 mg L^−1^ in a phosphate buffer pH 8.0 and incubated for another two hours at room temperature. The plates were washed using PBST and blocked for one hour using 1 g L^−1^ cysteine in 1 M NaCl and 0.1 M sodium acetate, pH 4.0. 

The binding of ochratoxin A-HRP conjugate in phosphate buffer pH 7.4 was performed at decreasing serial dilutions and incubated with the immobilized peptides for 1.5 h. The zero reference was determined with ochratoxin-HRP alone on 0.1 M ethanolamine blocked wells without immobilized peptide. All incubations were performed at room temperature. Detection was performed, after washing the microtiter plates using PBST, with the chromogenic HRP-substrate TMB in ready-made solution containing hydrogen peroxide. The absorbance was read after 20 min at 450 nm. 

### 3.4. SPR Testing for Binding Interaction

The binding interaction analysis of the peptide receptor with ochratoxin A was carried out on a CM5 (carboxymethylated dextran) sensor chip at 25 °C. HBS-EP (0.01 M HEPES pH 7.4, 0.15 M NaCl, 3 mM EDTA, 0.005% Surfactant P20) was used as the running and dilution buffer. Peptides (100 mg L^−1^ in 10 mM acetate buffer, pH 4.5) were immobilised using 0.4 M *N*-(3-dimethylaminopropyl)-*N*'-ethylcarbodiimide (EDC) and 0.1 M *N*-hydroxysuccinimide (NHS), applying amine coupling chemistry. In brief, a 1:1 mixture of NHS and EDC was injected as 5 μL min^−1^ flow rate for 7 min, followed by injection of 75 µL peptide ligand (5 µL min^−1^) for 15 min and blocking of residual binding sites with 1 M Ethanolamine, pH 8.3 (35 µL). To establish binding kinetics, various concentrations of ochratoxin A-BSA conjugate (0.01–100 mg L^−1^, 60 µL in HBS-EP pH 7.4) were injected at 5 µL min^−1^ over each immobilised peptide surface. BSA alone was injected as non-specific analyte control. Dissociation was monitored during 10 min without dissociating agents (flowing only running buffer). The kinetic parameters of the binding reactions were determined using BIAevaluation 3.2 software [46]. 

## 4. Conclusions

In this study, the application of computational modelling was employed to design a peptide ligand that binds to Ochratoxin A. A synthetic peptide ligand for ochratoxin A was designed using an innovative computational approach. The initial argument of the approach to simulate nature’s evolution by *de novo* designing a peptide ligand for a small molecular weight toxin was investigated using binding interaction analysis. The binding interaction of both peptides to ochratoxin A was confirmed in *in vitro* binding assays, and the affinity of the interaction was established using Biacore (SPR) analysis. Both peptide ligands showed weak affinity, indicated by fast on and off rates, and no need for surface regeneration. The 13-mer showed faster on and off rates when immobilized. The sensorgrams correlate well with the *in* *silico* data obtained with both LeapFrog and FlexiDock simulations. The results have shown the clear advantages of designing a peptide ligand for ochratoxin A *in silico*, which is a time-efficient, cost-effective production without exposure to toxic materials or the use of biological or animal resources. Using this approach, several peptides for ochratoxin A were successfully designed and synthesized, and their binding capacities were confirmed *in vitro*. It is also anticipated that the peptide ligands can be used in binding assays and affinity sensors. Further work will need to be conducted to characterise the affinity interaction between the peptides further and conduct a cross-interaction study.
